# Assessing potential future urban heat island patterns following climate scenarios, socio-economic developments and spatial planning strategies

**DOI:** 10.1007/s11027-015-9646-z

**Published:** 2015-03-27

**Authors:** Eric Koomen, Vasco Diogo

**Affiliations:** Department of Spatial Economics/SPINlab, VU University Amsterdam, De Boelelaan 1105, 1081 HV Amsterdam, The Netherlands

**Keywords:** Climate change, Land use change, Scenarios, Urban development, Urban heat island

## Abstract

Climate change and urban development will exacerbate current urban heat island effects. While most studies acknowledge the importance of projected temperature increases for raising urban temperatures, little attention is paid to the impacts of future changes in urbanisation patterns. Yet, steering urban development may be an effective strategy to further limit increases in the intensity and spreading of the urban heat island effect. We describe a method that allows exploring the impact of urban development scenarios on the urban heat island effect. This paper starts with a basic analysis of the strength of this effect in a temperate climate under relatively favourable conditions based on data from amateur weather stations and own observations. It explains local variation in observed temperatures and quantifies how the urban heat island effect may develop in the coming 30 years. Using the obtained relations, we assess potential future changes building on existing scenarios of climatic and socio-economic changes and a land use simulation model. Our measurements for the Amsterdam region in the Netherlands indicate that the urban heat island effect induces maximum temperature differences with the surrounding countryside of over 3 °C on moderately warm summer days. The simulations of potential future changes indicate that strong local temperature increases are likely due to urban development. Climate change will, on average, have a limited impact on these changes. Large impacts can, however, be expected from the combination of urban development and potentially more frequent occurrences of extreme climatic events such as heat waves. Spatial planning strategies that reduce the lateral spread of urban development will thus greatly help to limit a further increase in urban heat island values.

## Introduction

On average, urban areas are hotter than their surrounding rural areas, forming an urban heat island (UHI). This effect has already been documented in the early nineteenth century for the city of London, UK (Howard 1820), and has been studied intensively since the seminal work of Chandler (1962), Oke (1973) and Landsberg (1981). Recent examples include the work of Hart and Sailor (2009), Giannaros and Melas (2012) and Heusinkveld et al. (2014). The heat island effect can lead to increased cooling costs in summer time and exposes city dwellers to increased mortality due to higher temperatures (Aström et al. 2011; Döpp 2011) and has, therefore, attracted the attention for urban planners (e.g. NYSERDA 2006; Svensson and Eliasson 2002). Moreover, due to climate change, this effect may become stronger and more apparent (Patz et al. 2005; Wilby 2003).

Differences in temperature between urban and rural areas are largely determined by the ability of the urban fabric and surface materials to capture, store and slowly release incoming solar radiation, implying that, on aggregate, more thermal energy is stored in urban areas and cooling after the evening transition is slower than in rural areas (Oke 1982, 1995). Buildings and surface materials generally have a lower albedo and higher heat capacity than, for example, vegetation. In addition, multiple reflections between buildings and roads occur before the non-absorbed solar radiation is reflected to space, a phenomenon that is sometimes referred to as an urban canyon and that can be characterised by the sky view factor (Oke 1981; Svensson 2004). High buildings can also pose friction to air circulation and decrease average wind speed, resulting in smaller and slower cooling of building and street surfaces. In areas with a low sky view factor (i.e. narrow streets and/or high buildings), cities will thus trap more heat when cooling down at night.

Furthermore, the lack of vegetation and surface moisture results in a reduction in evapotranspiration, thus leaving more thermal energy available for heating the city (Akbari et al. 2001; Jusuf et al. 2007). Waste heat production and greenhouse gas emissions from anthropogenic activities (e.g. industry, car traffic, air conditioning) also contribute to increased heat radiation and increasing air temperatures in the cities (Hinkel and Nelson 2007; Sailor and Lu 2004).

Local morphological characteristics of urban areas such as building density and building height to width ratio have shown to contribute to the intensity of the UHI effect, by influencing heat release convection and advection in the urban boundary layer (Bottyán and Unger 2003; Hamilton et al. 2013; Hart and Sailor 2009; Svensson 2004). In addition, sprawling urban forms and nearby conurbations can also contribute to the build up of heat by limiting the ability for wind and turbulence to dissipate heat in the core of the urban zone (Bohnenstengel et al. 2011).

With changing climate conditions and an ever increasing urban population (Montgomery 2008; UN 2005), it is clear that the UHI effect will increase in the future. Yet, few studies exist that downscale these global changes to expected localised impacts. This paper describes a method that allows exploring the combined impact of urban development and climate change projections on the UHI effect. It starts with a basic analysis of the magnitude of the UHI effect under relatively favourable conditions in a temperate climate (Amsterdam, the Netherlands) and attempts to explain spatial variation in the observed temperatures. This analysis does not try to provide an exhaustive account of UHI formation; instead, we aim to capture the most important aspects for this process in our case study area to be able to create a quantitative assessment of the potential changes in the magnitude and spatial pattern of the UHI effect in the coming 30 years as a result of projected climatic and socio-economic changes. To assess potential future changes, we build on existing scenario studies and a land use simulation model. Using observed relations between average maximum daily temperatures and observed local UHI values, we are able to estimate the impact of global climate change on local UHI values. The land use change model allows the translation of macro-level socio-economic changes into potential future urbanisation patterns and thus the assessment of increased urbanisation on UHI values. This approach allows policy makers to assess the possible impacts of different spatial planning strategies on changes in urban temperatures.

## Methodology

### Analysing current UHI patterns

We describe current UHI patterns based on two separate analyses. Temporal variation in urban temperatures is described based on local temperature measurements derived from amateur weather stations, while spatial variation is measured along a route using mobile measurement devices and then explained using regression analysis and spatially explicit explanatory variables. For both analyses, we focus on the formation of UHIs on days with relatively favourable weather conditions to be able to assess the contribution of the most important factors on the intensity and spatial pattern of this phenomenon. The implications of selecting only favourable days on the robustness of our results are further elaborated upon in the discussion section

#### Temporal variation in urban temperatures

**Fig. 1:**
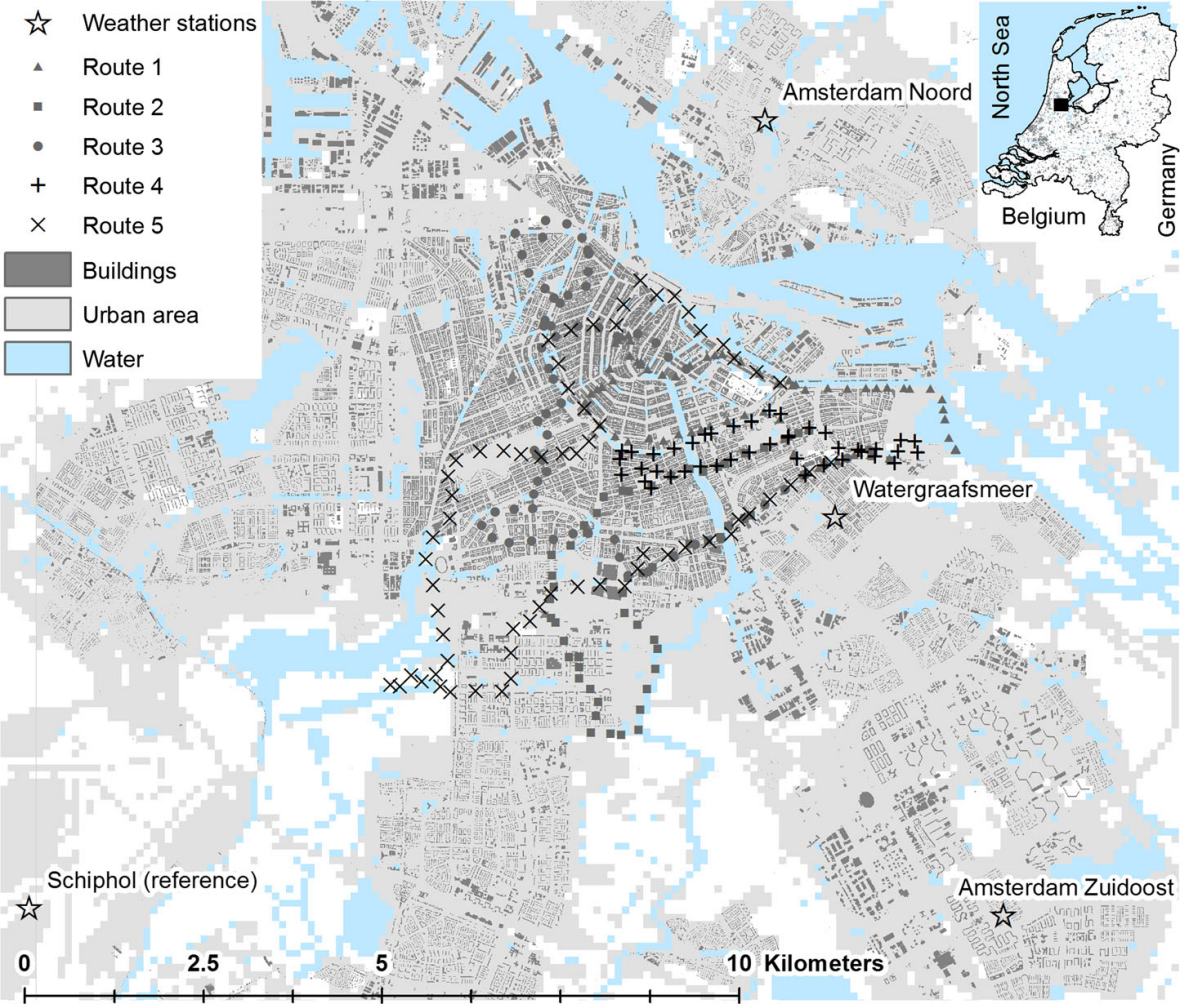
The Amsterdam study area including the location of the amateur and reference weather stations and measurement points along the bicycle routes. The figure also shows the two spatial variables (buildings and urban area) that were used in the explanatory analysis at their initial (finest) resolution

The spatial characteristics of urban areas trigger the formation of UHIs, but the intensity of the effect is also influenced by weather conditions such as temperature, wind speed and cloud cover that vary on a daily basis (Arnefield 2003; Tarleton and Katz 1995; Todhunter and Terjung 1990). To obtain a better understanding of temporal variation in UHI intensity and its relation to local weather conditions, we analyse hourly records of air temperature from five amateur-run automatic weather stations within the urban area of Amsterdam. Although amateur stations are not fully compliant with the standards of the World Meteorology Organization, they offer the possibility to study long-term temporal weather data in urban areas that lack official meteorological stations (Steeneveld et al. 2011). Urban temperatures were analysed for a 31-day period in the summer of 2010 (June 15 and July 15). This period was chosen because it includes substantial variation in temperatures (daily maxima in Amsterdam ranging between 13 and 33 °C) and it is—on average—characterised by conditions that favour UHI formation. After inspecting the data for all available amateur stations, we selected three stations that offered a consistent, complete time series for the same period. One station was discarded, as it offered a conspicuous afternoon peak in registered temperatures that is likely due to direct sunlight, and another station could not be used as its time series was incomplete. Table 1 describes the basic characteristics of the selected stations that were hitherto not included in any analysis of urban temperatures, and Fig. 1 shows their locations.

**Table 1:** Amateur weather station characteristics and their UHI_max_ values averaged over days with favourable conditions at the reference station (*N* = 19)

Weather Station (ID)	Latitude	Longitude	Elevation (m)	Average UHI_max_
Amsterdam Noord (MC9433)	52.40	4.92	6	3.3
Amsterdam Zuidoost (INOORDHO32)	52.30	4.97	3	3.0
Watergraafsmeer (INOORDHO97)	52.35	4.93	1	3.5
Schiphol Airport (EHAM)	52.30	4.77	2	reference station

Elevation is specified relative to ground level. Variation in absolute elevation (referring to NAP: Amsterdam Ordnance Datum) in the Amsterdam region is minimal and ranges from around −5 to +2 m

To characterise the heat island effect, we calculated the maximum UHI intensity (UHI_max_) defined as the maximum difference in hourly temperatures between the urban stations and the nearby reference station of Schiphol observed during a 24-h period. The reference station of Schiphol airport is operated by the Royal Netherlands Meteorological Institute (KNMI) and located close to Amsterdam at about 10 km from centre of the city in an open non-urban environment. It also captures the local near-maritime climate conditions of Amsterdam, as it is equally far from the coast line (approximately 20 km).

**Fig. 2:**
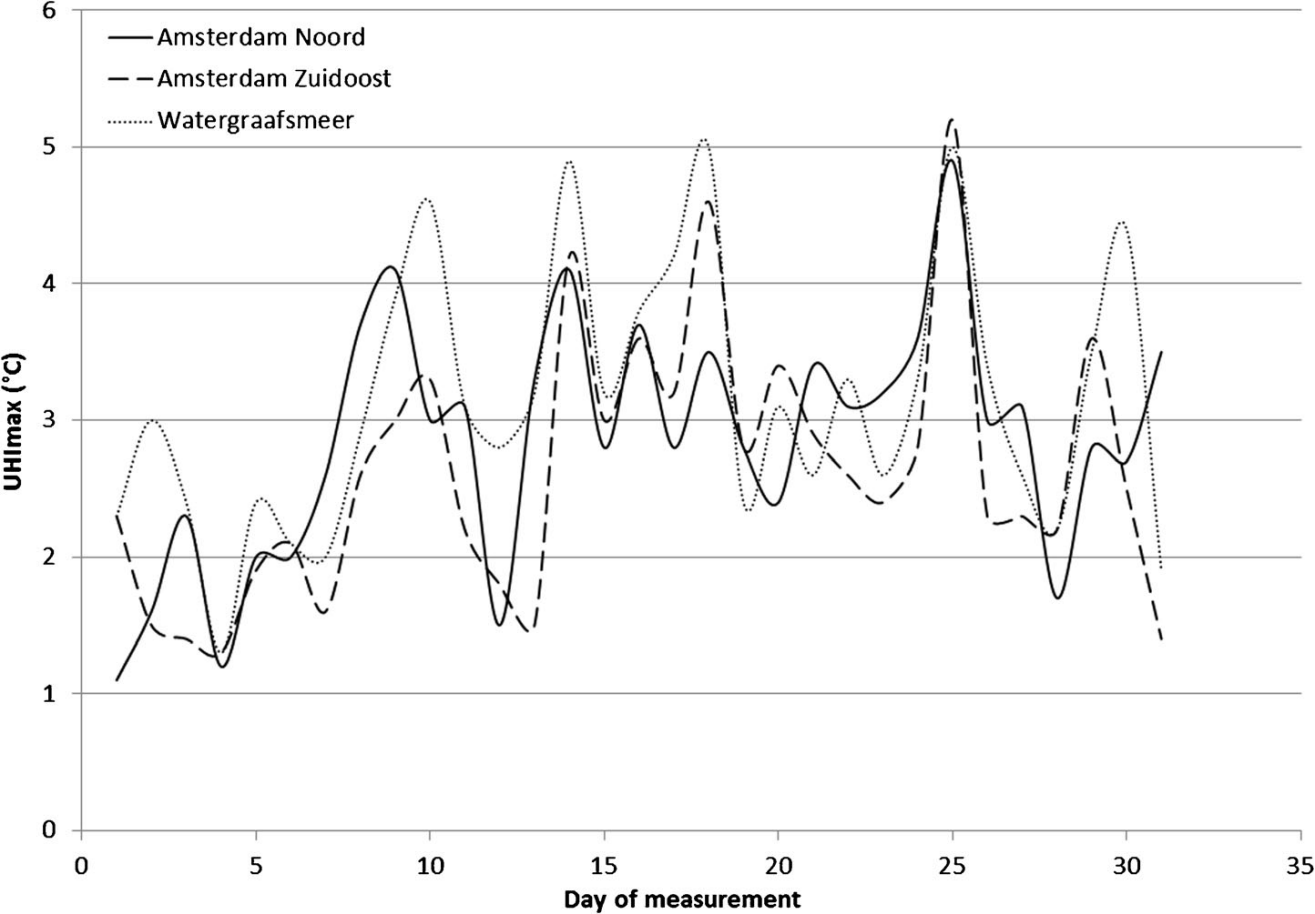
Observed daily maximum urban heat island values (UHI_max_) at three amateur weather stations during a 30-day period in the summer of 2010 (June 15 to July 15)

The amateur stations show consistent temperature differences with the rural reference station, and their daily UHI_max_ values range between 1.2 and 5.2 °C for the complete studied period (Fig. 2). The temporal variation in UHI_max_ values indicates the importance of daily differences in climatic conditions. We analysed the relations between UHI_max_ values observed at the urban amateur weather stations and several key variables describing the daily weather conditions at the Schiphol reference station. The results of this analysis are included in Appendix and confirm our expectations founded in prior research (e.g. Arnefield 2003; Steeneveld et al. 2011; van Hove et al. 2011): UHI_max_ increases with increasing daily maximum temperature and sunshine duration and decreases with increasing wind speed. Especially wind speed is known to have a strong inverse relation with the heat island effect; speeds in excess of 4 m/s already substantially decrease the effect (Giannaros and Melas 2012). Daily maximum temperature provides the single most important contribution to explaining variation in UHI_max_ values in our analysis. To test the impact of favourable conditions on this relation, we repeated the analysis for those days with favourable conditions that we defined as follows: low daily mean wind speed (<4.5 m/s), abundant sunshine (percentage of maximum potential sunshine duration ≥35 %) and minimal precipitation (daily precipitation amount <0.5 mm). The combination of these conditions applied for 19 out of the 31 selected days and provides a fairly similar result: the observed temperature difference between urban and rural areas increases by about 0.09–0.17 °C for each degree increase in maximum daytime temperature (Appendix). The observed relations do not describe all observed UHi_max_ variation per station, as other factors will play a role too. Although average daily weather conditions were fairly favourable during the observation period, some hourly variation will exist in, for example, local weather conditions at the moment when the heat island effect is building up. Higher wind speeds in the evening hours may limit UHI values on particular days. Likewise, local weather differences between the city of Amsterdam and the nearby reference station at Schiphol (e.g. in cloud cover or humidity) may influence these values. The specific setup of the amateur stations will also influence daily UHI_max_ values^1^ as will errors in measurement. The more structural differences between the stations may be due to differences in local conditions of the measuring devices (e.g. in exposure and elevation) or the local degree of urbanity.

Building on this assessment of temporal variation in the UHI effect, the next section describes our analysis of spatial variation in urban temperatures.

#### Spatial variation in urban temperatures

**Fig. 3:**
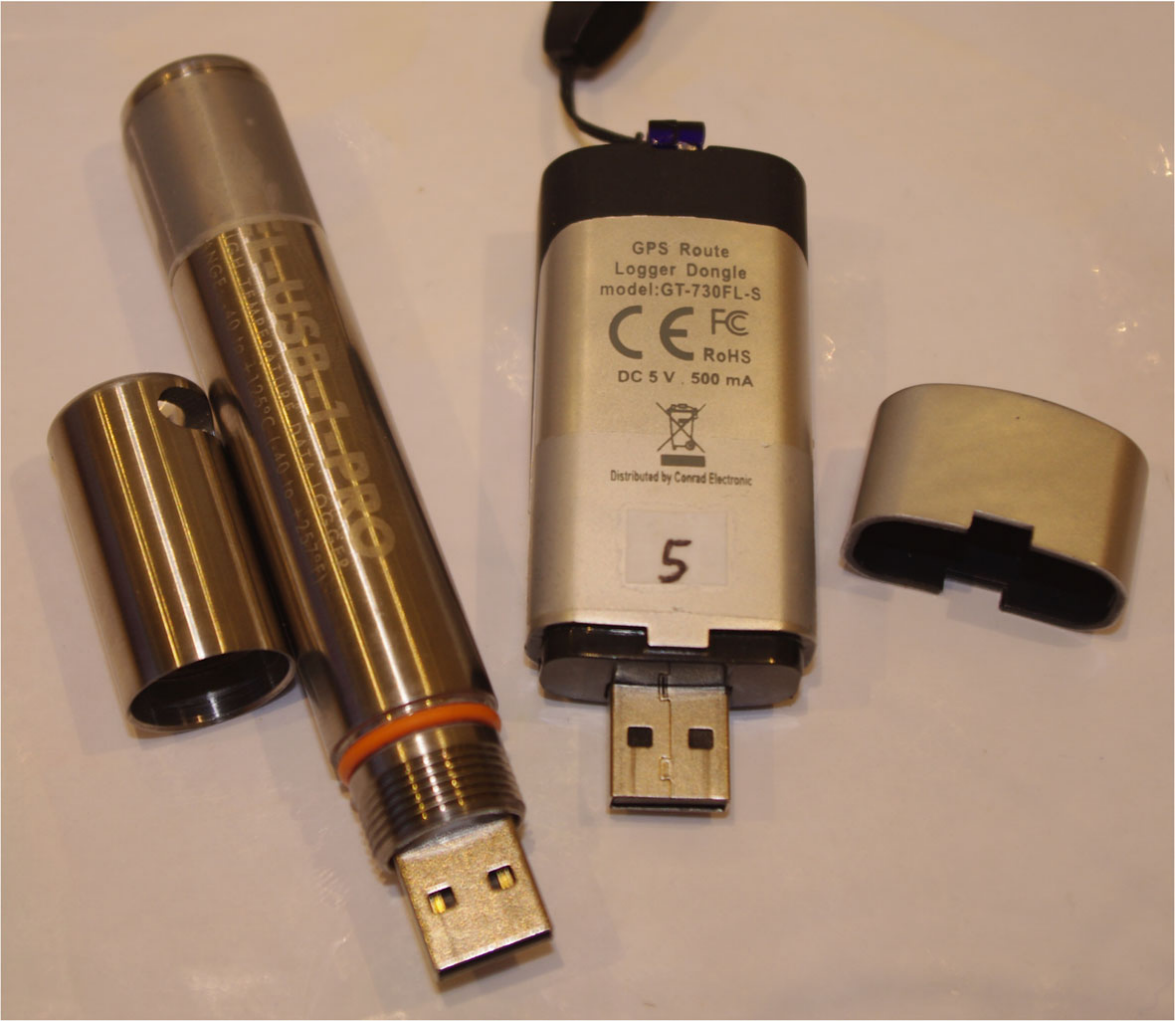
Photographs showing the measurement devices used during the bicycle runs

Local urban temperatures were measured using small portable position and temperature data loggers fixed to bicycles while travelling along five circular routes through the city of Amsterdam. The key specifications of the applied devices are listed in Table 2, while pictures of the devices are included in Fig. 3. We consider the accuracy of these instruments sufficient for our purpose and comparable to similar measurements reported by others (Brandsma and Wolters 2012; Giannaros and Melas 2012; Heusinkveld et al. 2010; Steeneveld et al. 2011).

**Table 2:** Key specifications of data logging devices applied in this study

Accuracy	GPS-based position logger	Temperature logger
Manufacturer	Canmore	Lascar
Model name	GT-730	EL-USB-1-Pro (4×)/EL-USB-2+ (1×)
Accuracy	Circular Error Probability (CEP) 5 m	Typical overall error ±0.2 °C/±0.5 °C
Logger resolution	0.000001 decimal degree (WGS84)	0.1 °C/0.5 °C

Device specifications are taken from the manufacturer’s documentation

The different routes were designed in such a way that they that reached open areas outside the city, various neighbourhoods with different spatial characteristics within the city and the historic centre. All routes started in the east of town, passed through the dense centre and less dense suburbs before reaching more open areas on the outskirts of town and returning to the east. Measurements were taken every minute during an approximate 2-h period after sunset (from around 22.00 to 0.00) on days with favourable conditions for the formation of UHIs in the months of June in 2012 and 2013. For selecting days with favourable weather conditions, the same criteria were applied that were introduced in the preceding section. The late evening period was chosen because UHI values are known to be highest after sunset when the heat stored in artificial surfaces is slowly released (Steeneveld et al. 2011; van Hove et al. 2011). The UHI effect is strongest in summer (ibid.), and the month of June is selected to represent Dutch summer conditions, while offering the chance of long sunny days. In total, we selected five measurement sets that were obtained on three different days. The selected days aim to capture the spatial distribution of the UHI effect in the study area under relatively favourable condition days for a wide range of maximum daily temperatures. These temperatures range from below average^2^ to extremely warm (see Table 3 for an overview of the basic meteorological conditions of the selected days, while Fig. 1 shows the observation points along the five different routes).

**Table 3:** Meteorological conditions at the reference station (Schiphol airport) during the observation days compared to average values for the three summer months in the 1980–2010 period

Date	Maximum temperature (°C)	Sunshine (% of daylight hours)	Precipitation (mm)	Wind speed range (21–24 h in m/s)	Number of routes
June 17, 2012	19.7	53	0	0.8–2.7	1
June 16, 2013	17.5	35	<0.05	0.6–2.1	1
June 18, 2013	29.0	54	0	2.6–3.5	3
1980–2010	21.1	42	2.5	Daily mean 4.3	

Sources: www.weergegevens.nl for day values, www.buienradar.nl for wind speed range at time of cycling and www.knmi.nl for Schiphol data 1980–2010 (average values for summer months calculated by authors)

For all measurement points along the routes, the UHI effect was described by linking the urban temperatures obtained at each minute to the logged position at that moment and comparing it to the temperature measured at the Schiphol Airport reference station. The latter are stored at 10-min intervals and were linearly interpolated to match the exact minutes of the observations.

The observed variation in the UHI effect was explained through linear regression analysis focussing on two essential causal factors: the weather conditions at the day of measurement and the spatial characteristics of the observation points. The weather conditions are described by the maximum daily temperature at the reference station, which was observed to positively influence UHI intensity (Sect. 2.1.1). Variation in other weather conditions was largely controlled for through our selection of relatively sunny, dry observation days with low wind speeds at the time of measurement. The description of variation in spatial characteristics focussed on describing the degree of urbanity around the observation points. We theorise that the UHI effect has a general component related to the different albedo of built-up surfaces and their tendency to store heat during the day and slowly release that in subsequent hours and a more specific component related to the layout of the urban fabric that limits the sky view of the surface (and thus the radiation of heat) as well as blocking wind flows that could help transport heat (Steeneveld et al. 2011). Both components are part of the local scale (one to several kilometres) of urban temperature measurements as distinguished by Oke (1984). We deliberately exclude the microscale level temperature variation that is associated with individual buildings or trees, as that is beyond the reach of our measuring method and research objectives. Neither do we want to focus on the much coarser mesoscale level (typically tens of kilometres) described by Oke, as we are especially interested in inner city variation in temperatures. To operationalise the more general component, we describe the average amount of urban surface (i.e. infrastructure, residential, commercial or associated land use in 2008 at 100-m resolution, taken from www.cbs.nl) surrounding the observations. The more specific urban fabric component is characterised by a data set describing the total volume of any type of building at a 100-m resolution. This description is aggregated from a fine-grained (5-m resolution) data set containing building heights. It captures the density and heights of buildings in the city and thus links closely to the sky view factor (for a more extensive discussion on the urban volume data set, see Koomen et al. 2009). The combination of a generalised land use data set and a detailed building volume data set was also applied to characterise the urban context in a neighbourhood-scale meteorological model that simulates temperatures for the London area (Hamilton et al. 2013). As current literature does not provide conclusive information on the exact spatial scale at which these variables act, we tested several spatial ranges. Using standard, moving window type of filter operations, we characterised the conditions in different-sized neighbourhoods around individual cells in a relatively fine 100-m grid, describing the average amount of urban area in eight neighbourhoods ranging from 100 to 10,000 m and the total amount of urban volume in four neighbourhoods ranging from 100 to 1000 m. These spatially explicit statistics offer a progressively smoother and generalised representation of urbanity in the study area and capture neighbourhood conditions while maintaining a relatively fine representation of the urban fabric. Table 4 provides descriptive statistics of the applied variables for the different neighbourhood ranges at which they were constructed. More specific spatial variables that were relevant for the explanation of local variation in urban temperatures in other studies (Bottyán and Unger 2003; Hart and Sailor 2009; Steeneveld et al. 2011) were also tested. These variables related to degree of sealed surface, availability of water as potentially cooling element and availability of specific types of green space such as wooded parks and open fields. They were discarded from the analysis, however, as they did not yield statistically significant results. This insignificance may partly arise from the correlation between the different variables that, to a large extent, depend on the local amount of built-up features. The local amount of sealed surface has a direct relation with the number of buildings in the direct vicinity, while the amount of green space complements the amount of built-up features in urban areas. Studies focussing on the explanation of local variation in urban temperatures thus tend to use either amount of vegetation or fraction built area (e.g. Brandsma and Wolters 2012; Heusinkveld et al. 2014). Water bodies have a more ambiguous impact. Due to their high heat capacity, they may limit warming in the early summer (when the water is still relatively cold), but they may suppress cooling in the evenings and later in summer as was recently explored by Steeneveld et al. for the city of Rotterdam, the Netherlands (2014). Also, in our case, the presence of water did not provide a consistent impact on urban evening temperatures.

**Table 4:** Descriptive statistics of the variables included in the explanatory analysis

Variable name	Units	Range	Mean (SD)
Urban heat island effect	°C	−0.1–3.4	2.2 (0.8)
Maximum temperature at reference station (one value per day)	°C	17.5–29.0	n.a.
Average amount of urban area within circular neighbourhoods of	ha		
100 m		0 or 1	0.858 (0.350)
200 m		0.000–1.000	0.859 (0.199)
500 m		0.160–1.000	0.870 (0.157)
1000 m		0.344–0.984	0.867 (0.120)
1500 m		0.506–0.968	0.853 (0.096)
2000 m		0.563–0.961	0.837 (0.088)
5000 m		0.584–0.819	0.767 (0.051)
10,000 m		0.451–0.633	0.565 (0.044)
Total amount of urban volume within circular neighbourhoods of	hm^3^		
100 m		0.000–0.302	0.090 (0.066)
200 m		0.000–1.145	0.381 (0.241)
500 m		0.000–5.674	2.418 (1.316)
1000 m		0.083–18.354	9.293 (4.443)

Statistics obtained for urban temperature observation locations only (*N* = 302), not for full coverage of the study area

To account for the fact that the impact of maximum temperature on UHI effects will only apply within urban areas, we cross the maximum temperature variable with the average amount of urban area surrounding an observation. This results in an assessment of the base level of urban heat at a particular day given a specific temperature. This level is higher on warm days as we found in our analysis of temporal variation in urban temperatures (Appendix). By definition, this augmented urban temperature should be zero when no urban area is present. Table 5 lists the outcomes for a range of spatial resolutions at which the average amount of urban area was obtained. The table shows that the explained variance in the regression analysis (as measured in *R* ^2^) increases with increasing size of the neighbourhoods around the observations for which the average amount of urban area is calculated. Apparently, our UHI measurements respond to gradual rather than very local changes in the amount of urban area in the surroundings. A remarkable outcome is that the constant in the regression becomes negative at larger distance ranges, whereas this should, by definition, approach zero as the non-built up areas around the city form the reference against which the urban temperatures are compared. This behaviour is probably caused by the limited amount of observations that are fully outside the sphere of urban influence. To account for that, we continue our explanatory analysis with a so-called no-intercept model that forces the regression through the origin. This approach is appropriate when the outcome of the dependent should be zero when the independent is zero and has been applied previously in studies explaining UHI intensity from the presence of urban features (Brandsma and Wolters 2012). We selected the 1000-m range for the average amount of urban area variable for the continuation of our analysis, as this is the range where the constant started to become negative. That the coefficient is insignificant and the *t* value relatively low also indicates that the constant contributes little to the explanation of UHI values at this distance range. Table 5, furthermore, lists the impact of adding the urban volume variable to the explanation. Again, we find that an increase in the radius used for aggregating local urban volume leads to an increase in explained variance. We, therefore, also select the 1000-m neighbourhood for this variable, as this spatial resolution seems to best match the spatial variation in our temperature measurements.

**Table 5:** Explanatory analysis of observed urban heat island values using maximum temperature at reference station (Ref_*t*_) crossed with different neighbourhood sizes for the urban area (UA) variable and, for the selected range of 1000 m, different neighbourhood sizes for the urban volume (UV) variable

Distance range	*R* ^2^	Variable	Beta (SE)	*t* value
Step 1: testing different distance ranges for calculation of average amount of urban area surrounding observations (UA_distance range_)				
100 m	0.183	Constant	1.439 (0.103)	13.912
		Ref_t_* UA_100_	0.036 (0.004)	8.184
200 m	0.458	Constant	0.460 (0.115)	3.994
		Ref_t_* UA_200_	0.082 (0.005)	15.919
500 m	0.506	Constant	0.134*(0.123)	1.087
		Ref_t_* UA_500_	0.097 (0.006)	17.547
1000 m	0.567	Constant	−0.201*(0.126)	−1.601
		Ref_t_* UA_1000_	0.113 (0.006)	19.805
1500 m	0.592	Constant	−0.355 (0.127)	−2.799
		Ref_t_* UA_1500_	0.122 (0.006)	20.844
2000 m	0.604	Constant	−0.401 (0.126)	−3.192
		Ref_t_* UA_2000_	0.126 (0.006)	21.401
5000 m	0.661	Constant	−0.759 (0.126)	−6.031
		Ref_t_* UA_5000_	0.157 (0.007)	24.175
10,000 m	0.556	Constant	−0.420 (0.139)	−3.020
		Ref_t_* UA_10,000_	0.189 (0.010)	19.394
Step 2: testing different distance ranges for calculation of total amount of urban volume surrounding observations (UV_distance range_)				
	0.945^a^	Ref_t_* UA_1000_	0.104 (0.001)	71.868
100 m	0.953^a^	Ref_t_* UA_1000_	0.091 (0.002)	39.864
		UV_100_	3.142 (0.452)	6.948
200 m	0.954^a^	Ref_t_* UA_1000_	0.087 (0.003)	34.020
		UV_200_	0.965 (0.125)	7.692
500 m	0.955^a^	Ref_t_* UA_1000_	0.083 (0.003)	29.423
		UV_500_	0.189 (0.023)	8.368
1000 m	0.957^a^	Ref_t_* UA_1000_	0.079 (0.003)	25.672
		UV_1000_	0.060 (0.007)	9.155

All coefficients are statistically significant at 0.01 level, unless otherwise indicated

*Not significant at 0.05 level

^a^Regression specifications without a constant (the no-intercept models) in which *R* ^2^ measures the proportion of the variability in the dependent variable about the origin explained by regression. As this *R* ^2^ cannot be compared with those for models which include an intercept, we also include the no-intercept model without the addition of the urban volume variable for reference purposes

The selected regression result can be used to map the spatial variation in UHI values for the study area as follows:

1 $$ UH{I}_t=0.079\times Re{f}_t\times U{A}_{1000}+0.060\times U{V}_{1000} $$

where

UHI_*t*_: is the expected local UHI value for a maximum temperature *t* at the reference station (Ref)
UA_1000_: is the average amount of urban area within a circular neighbourhood of 1000 m, and
UV_1000_: is the urban volume within circular neighbourhoods of 1000 m.

On a dry, sunny day with low wind speeds and a maximum temperature of 20 °C at the reference station, this approach implies a maximum heat island effect of around 2.6 °C in the centre of the city where the maximum values for average amount of urban area and total amount of urban volume coincide. According to this analysis, this maximum effect increases to about 3.4 °C for days with a maximum temperature of 30 °C. These values match well with the observed UHI values from the amateur stations (Table 1) and bicycle runs (Table 4). Section 3 shows the estimated spatial variation in current UHI values following the above formulation. This basic explanatory model can also be used to assess potential future changes in a straightforward way without requiring many additional assumptions as is discussed in the following subsection.

### Simulating future UHI patterns

#### Future climate scenarios

The UHI effect is likely to become stronger in future as both average temperature and amount of urban area are expected to increase. A comprehensive set of climate change scenarios for the Netherlands was made available by the Royal Dutch Meteorological Institute (Van den Hurk et al. 2006) using the best available information on climatic changes, uncertainties and spatial variation available at that time. This was the most recent set of regionally downscaled scenarios available at the time of analysis. The scenarios do not link directly to the well-known emission scenarios originally published by the United Nations Intergovernmental Panel on Climate Change (IPCC 2000) but, instead, follow the two main uncertainties in climate change in Western Europe that relate to changes in temperature and prevailing air circulation patterns. The temperature uncertainty relates to an increase of either 1 or 2 °C in the average yearly global temperature for 2050, whereas the uncertainty in circulation pattern refers to changed or unchanged air circulation above Europe. In combination, the two uncertainties result in four climate scenarios that are referred to as moderate (for the 1 °C increase in temperature, abbreviated to G) and warm (W or the 2 °C increase), with or without a ‘+’ indicating a change in air circulation pattern. Table 6 lists the key characteristics of these national scenarios for average summer conditions in the 30-year climate period around 2050 downscaled for the De Bilt weather station at approximately 40 km from Amsterdam (the nearest station for which such projections are available). The expected average maximum temperatures in summer time are used to describe possible future urban heat conditions applying the observed relations described in Eq. 1. The table also shows that, especially in the W scenarios, the number of hot and warm days is likely to increase strongly, which will result in a more frequent occurrence of strong UHI effects. The amount of rain and frequency of rainy days are not expected to increase much, so there is little chance for extra cooling by increased humidity or cloud cover.

**Table 6:** Current and projected summer conditions according to the four Dutch climate scenarios downscaled for the De Bilt weather station at approximately 40 km from Amsterdam

	Present 1976–2005	G scenario ±2050	G+ scenario ±2050	W scenario ±2050	W+ scenario ±2050
Average day temperature (°C)	16.8	17.7	17.6	17.9	19.6
Average maximum temperature (°C)	21.7	22.6	23.1	23.4	24.5
Nr. warm days (max. temp. ≥25 °C)	24	30	34	39	47
Nr. hot days (max. temp. ≥30 °C)	4	7	9	10	14
Total precipitation (mm)	214	220	193	227	173
Days without rain (%)	51	52	57	54	61

Source: http://www.knmi.nl/klimaatscenarios. More information is offered by Van den Hurk et al. (2006)

#### Future urban patterns according to socio-economic scenarios

To provide an outlook on future urban patterns, we apply a land use simulation model that is well-established in spatial planning and climate adaptation research in the Netherlands and several other countries: Land Use Scanner (Hoymann 2010; Koomen and Borsboom-van Beurden 2011; Kuhlman et al. 2013; Te Linde et al. 2011). This GIS-based model is rooted in discrete choice theory (McFadden 1978) and integrates sector-specific inputs (e.g. regional demand for residential land) from other dedicated models. It simulates the choice between mutually exclusive uses (e.g. residential, commercial, agricultural, recreation) for a particular location. This choice depends on the demand-supply interaction for land, with sectors competing within suitability and policy constraints. In practical terms, it combines the demand for land for various societal functions specified for different regions (and derived from specialised models run by different research institutes) with a grid-cell-based definition of local suitability for these different types of use at a 100-m resolution. Details on the applied allocation algorithms, calibration and validation of the model can be found in other publications (Koomen et al. 2011; Loonen and Koomen 2009). To reflect the inherent uncertainty in future societal conditions and incorporate alternative spatial planning strategies, we have selected the two most diverging scenarios from an existing Dutch scenario study (CPB et al. 2006). The Global Economy (GE) scenario is part of the A1 scenario family in the IPCC terminology and shows strong population and economic growth in combination with a liberal government that poses little restrictions to urban development. In the Regional Communities (RC) scenario (based on the B2 scenario family of IPCC), the population remains more or less stable, with modest economic growth and a more strict government that safeguards public goods such as historic open landscapes and natural areas. In relation to urbanisation patterns, the scenarios oppose large-scale dispersed urban development (GE) with limited and more compact extension. These two scenarios thus highlight different pathways for future development that depend on the absence or presence of urban containment policies. As such, they confront policy makers with the potential consequences of their spatial planning alternatives. Details on the implementation of the two scenarios into the Land Use Scanner model are provided elsewhere (Dekkers et al. 2012).

Based on the simulated land use patterns for 2040, we created two updated versions of the urban volume data set, one for each scenario. These were created according to the following rules: (1) for locations where land use did not change between 2008 (base year for simulation) and 2040, the urban volume values for the base year were maintained, and (2) for locations that became urbanised in the simulation period, the urban volume value was updated to the mean urban volume value observed in the base year within built-up areas. Subsequently, the simulated urban area patterns for 2040 and the updated urban volume data layer were used to create new data layers describing the urban area and urban volume conditions in 1000-m neighbourhoods (UA_1000_ and UV_1000_ as specified in Eq. 1) for 2040. In combination with the expected average maximum summer temperatures, these were used to depict spatial variation in the future UHI effect. Since the climate scenarios relate to average weather conditions in the 2035–2065 period, we believe that we can combine them with our land use simulation results related to 2040.

## Simulation results

**Fig. 4:**
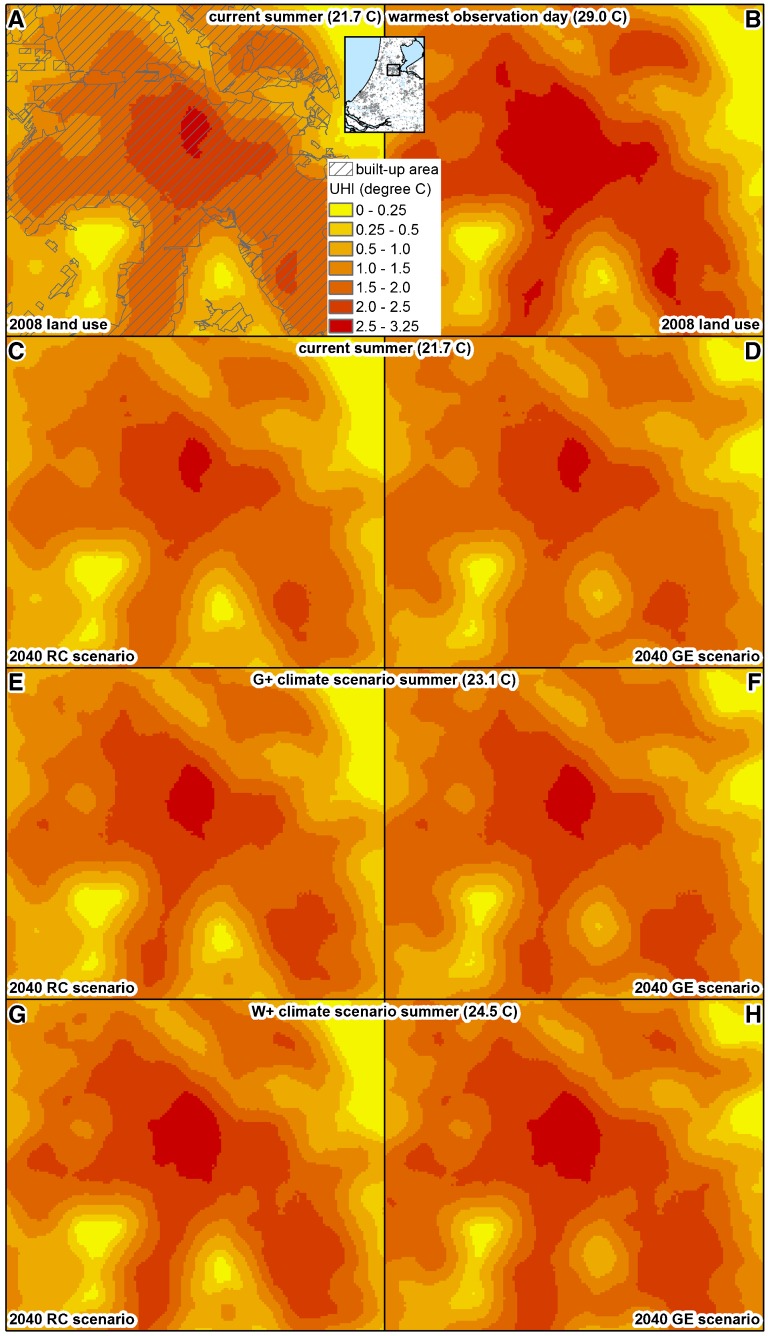
Overview of simulated urban heat island patterns for current land use (maps **a**, **b**) and scenario-based land use simulations combined with different maximum temperatures associated with average summer conditions in current (**c**, **d**) and future climate scenarios (**e**, **h**)

Using the statistical relations obtained in our explanatory analysis of local measurements of spatial variation in the UHI effect and data sets describing current urban area and urban volume patterns in Amsterdam, we mapped current spatial variation in the UHI effect for the entire city as depicted in Fig. 4a, b. These figures are based on the current average maximum temperature in summer in the Netherlands (21.7 °C, see Table 6) and the relatively high temperature (29.0 °C) of one of our observation days. The inner city is clearly distinguishable with values of over 2.5 °C on an average summer day and close to 3.2 °C on the warmest day of our observations. The difference between these days is not so much in the maximum values of the UHI effect, but rather in its spatial distribution. Moving towards the outskirts of town, the temperature shows a gradual decrease. In the areas surrounding the old centre, with lower urban densities, the UHI effect is found to be between 1.5 and 2.5 °C. Still further from the city centre, the UHI pattern becomes more heterogeneous, with several areas with moderately high UHI values corresponding to the suburban lobes of Amsterdam alternating with low UHI values for the open areas between the lobes. A second area with high UHI values represents a dense commercial district in the south-east of the city.

The simulated future UHI patterns are shown in Fig. 4c–h. These maps are based on the simulated extension of the urban area in combination with three different maximum temperatures: the current average maximum temperature in summer and the expected maximum temperatures in summer for two climate scenarios: G+ and W+ (as described in Table 6). From these maps, it can be observed that the change in UHI differs per scenario. The RC scenario shows a small concentrated UHI increase in high urban volume value areas in the centre, whereas the GE scenario shows a more dispersed spread of the UHI effect. This follows from the stronger focus on concentration of activity in the RC scenario, while the GE scenario allows more urban development at the edges of town. The increases in temperature following the climate scenarios result in more extreme UHI values with maximum UHI values in the centre rising to about 3.25 °C and spreading over larger areas. On hot summer days that are expected to occur more frequently, the UHI effect will be much stronger.

## Conclusions

Our measurements for the Amsterdam region in the Netherlands show that the UHI effect induces maximum temperature differences with the surrounding countryside of over 3 °C on moderately warm summer days with an average maximum daytime temperature and up to 5 °C on warm days. These values are in line with recent studies into the UHI effect that made use of amateur weather stations in several Dutch cities and that found mean daily maximum values of 2.3 °C and extreme values of 4–6 °C for specific days (Steeneveld et al. 2011; Wolters et al. 2011). Another recent study for the 2006 heat wave found night-time air temperatures in Amsterdam to be 7–9 °C higher and surface temperatures more than 10 °C higher than in the rural surroundings (Van der Hoeven and Wandl 2013). Besides their specific focus on a heat wave, these higher values may also be related to their reliance satellite imagery, a technique that has difficulty with distinguishing surface temperature from the overlying air temperature that is actually measured (Mavrogianni et al. 2011). Other studies using portable or miniature near-surface air temperature logging devices for cities in different countries documented UHI values that are very similar to our results: 3–5 °C for Rotterdam, the Netherlands (on a day with a maximum temperature of 30 °C, see Heusinkveld et al. 2014; van Hove et al. 2011), 3–5 °C on particularly warm days (35–40 °C) in Portland Oregon (Hart and Sailor 2009) and 2–3 °C for the typically warm summers in Thessaloniki, Greece (Giannaros and Melas 2012). So we believe that the our measurements provide a reasonable account of the magnitude of the UHI effect in Amsterdam under typical summer conditions that favour the development of this effect and that have maximum daily temperatures in the range of about 20–30 °C. An analysis of the frequency of occurrence of favourable conditions for UHI formation at Schiphol airport during the three summer months (June, July and August) in the past 30 years (1980–2010) indicates that the combination of these conditions (wind speed <4.5 m/s and percentage of maximum potential sunshine duration ≥35 % and daily precipitation amount <0.5 mm) held true for 31.5 % of the summer days.^3^ Under less favourable conditions, the UHI will be much less pronounced or even absent, whereas on winter days, relations may be different due to lower solar intensity, shorter days, more abundant shadows and possibly also more anthropogenic influences (e.g. from traffic or heating of houses). A separate analysis would thus be required for assessing this seasonal variation (cf. Svensson and Eliasson 2002). Such an analysis could be used to more specifically establish possible consequences of the UHI effect in terms of limiting the need for heating in urban areas in winter time. Accounting for UHIs on (prolonged periods of) extremely warm days would also require additional research, as would the interaction of urban temperatures with other weather-related health issues such as smog and air pollution.

In our explanatory analysis of the observed spatio-temporal variation in UHI values, we combine the spatial variation in degree of urbanity that was encountered during the different bicycle routes with the temporal variation in temperatures at the days of cycling. This temporal variation is included by referring to the daily maximum temperature following the relatively strong relation that we found between these temperatures and the maximum heat island values observed at various amateur weather stations in the city. We find that the degree of urbanity matters especially within relatively large distance ranges of about 1000 m. This finding is in line with recent studies into the spatial variability of the UHI in the Netherlands that use spatial distance ranges of 400 m (Brandsma and Wolters 2012), 600 m (Steeneveld et al. 2011) and 700 m (Heusinkveld et al. 2014). These results indicate that the UHI effect is dependent on the amount of urban area and presence of building in a fairly large radius, implying that strategies to mitigate increased urban temperatures should foremost focus on limiting the further expansion of large urban areas. Our simulations of the future UHI patterns based on different urbanisation patterns indicate the impact of limiting urban expansion and hint at the benefits of containing urban growth.

While measurements of the UHI effect have become fairly abundant in recent years, explanatory studies are much scarcer and assessments of the combined impact of climatic and socio-economic changes on the future state of urban temperatures are virtually absent. A notable exception is offered by the urban meteorological models applied to the greater London area to simulate the current urban climate and likely changes related to the large urban development for the Olympic Parkland (Bohnenstengel et al. 2011; Hamilton et al. 2013). Yet, our approach is different, as it combines projected changes in urbanisation patterns and maximum day temperatures from socio-economic and climate scenarios in a straightforward way without requiring full-fledged meteorological models. The reliance on limited amounts of data makes our approach applicable in quick, initial assessments of potential future changes in the local UHI effect. In addition, it allows assessing the relative importance of socio-economic and climatic changes for this effect. In our case study for the city of Amsterdam, we found that climate change increases the intensity of the UHI effect, while urban development leads to strong local temperature increases as well as causing larger areas to be affected. An increase in average maximum temperature in summer of 1–3 °C as projected in Dutch climate scenarios for 2050 will only lead to a marginal increase in urban temperatures of about 0.1–0.3 °C for inner urban locations. The UHI effect reacts stronger on the day-to-day variation in summer temperatures. The more frequent occurrence of warm or hot days that is also projected in the climate scenarios will have a larger impact on future UHI values. Even larger impacts can be expected from the combination of urban development and more frequent occurrences of extreme climatic events such as heat waves. The presented simulation approach allows policy makers to explore the potential consequences of different urbanisation strategies. It is able to show the benefits of compact urbanisation in limiting the lateral extent of the UHI effect as well the trade-off with increasing urban temperatures that result from adding residences to the existing urban fabric instead of to open spaces around cities. The quantitative nature of our approach distinguishes it from other spatial-planning-oriented tools that map urban climatic conditions in a more qualitative way (Ren et al. 2012).

Our approach focuses on the local (neighbourhood) scale of urban temperatures as distinguished by Oke (1984), implying that microscale temperature differences related to individual buildings or trees are not explicitly measured. The perceived importance of such very local differences seems to depend on the moment and method of measurement (compare, for example, Aniello et al. 1995 with Brandsma and Wolters 2012). Yet, much research focuses on defining mitigation measures that may help limiting microscale variation in urban temperatures, proposing, for example, an increase in vegetative cover and the application of higher albedo surface materials (Solecki et al. 2005) or the provision of small green spaces or water bodies (Kleerekoper et al. 2012). To assist the definition of this type of mitigation strategies and assess their potential impacts, more detailed measurements of urban temperatures are required that focus, amongst others, on the presence of individual trees or water bodies.

## Figures and Tables

**Fig. 5:**
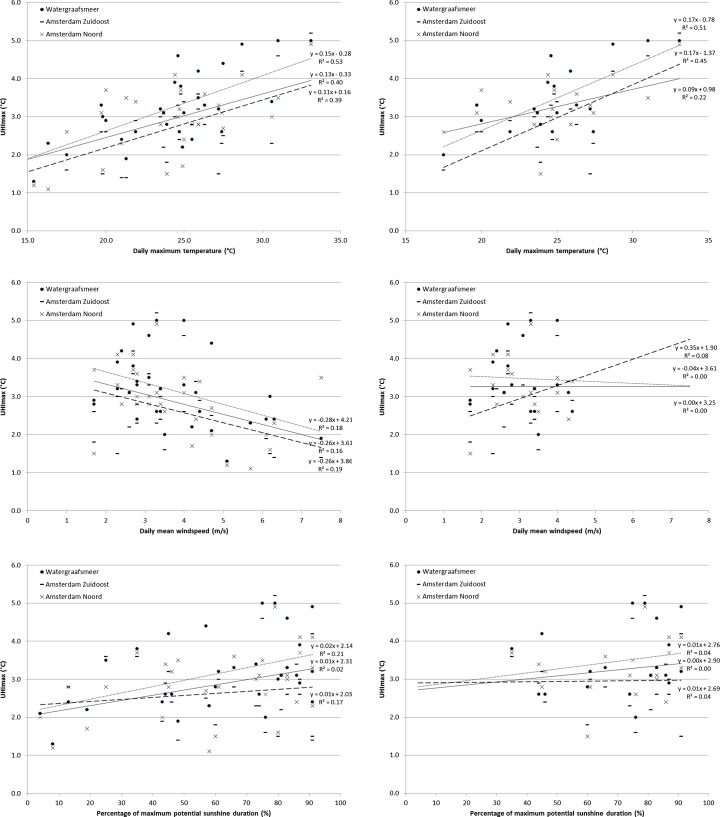
Variation in UHI_max_ related to daily weather conditions for full study period (*left*) and selection of days with conditions (*right*). The *trend lines* apply the same line styles as Fig. 1

## Appendix UHI_max_ variation related to daily weather conditions

This appendix shows the relations between UHI_max_ observed at the urban amateur weather stations and daily weather conditions observed at the Schiphol reference station. The figures on the left refer to the full study period of 31 days and show the expected relations: UHI_max_ increases with increasing daily maximum temperature and sunshine duration and decreases with increasing wind speed. The figures on the right only contain the days with favourable conditions for urban heat island formation (low wind speed, abundant sunshine, limited precipitation). The exact selection criteria for these days are discussed in the paper. The regression results included in the figures show that daily maximum temperature provides the single most important contribution to explaining variation in UHI_max_ values. After applying the criteria for selecting the favourable days for urban heat island formation, the remaining variation in wind speed and sunshine duration fails to contribute to explaining variation in UHI_max_. The applied criteria thus act as they should: they take out the days in which UHI formation is limited by non-favourable conditions. The relation with daily precipitation is not shown here, as rain was absent on all but 5 days. Rainfall was used as an exclusion criterion as it is expected to influence the heat balance in the city.

## Footnotes

1 The specific urban setting of the amateur station may assert a different influence on temperature development from day to day, for example, when wind from certain directions is blocked.

2 The 30-year average maximum daily summer temperature of the reference station is 21.1 °C (see Table 3).

3 The frequency of occurrence of the specified conditions was calculated using a dataset describing daily weather conditions provided by the Royal Netherlands Meteorological Institute (www.knmi.nl/klimatologie/daggegevens/download.html). The individual conditions held true for 57 % (wind speed) and 58 % (solar radiation) and 62 % (precipitation) of the summer days.
